# Anti-Inflammatory Effect of Fluvastatin on IL-8 Production Induced by *Pseudomonas aeruginosa* and *Aspergillus fumigatus* Antigens in Cystic Fibrosis

**DOI:** 10.1371/journal.pone.0022655

**Published:** 2011-08-03

**Authors:** Stéphane Jouneau, Mélanie Bonizec, Chantal Belleguic, Benoit Desrues, Graziella Brinchault, Jeanne Galaine, Jean-Pierre Gangneux, Corinne Martin-Chouly

**Affiliations:** 1 EA 4427 Signalisation et Réponse aux Agents Infectieux et Chimiques, Université de Rennes 1, Institut de Recherche Santé Environnement Travail, Institut Fédératif de Recherche 140, Rennes, France; 2 Service de Pneumologie; 3 Service de Parasitologie-Mycologie, Hôpital Pontchaillou, Centre Hospitalier Universitaire de Rennes, Rennes, France; Centre de Recherche Public de la Santé (CRP-Santé), Luxembourg

## Abstract

**Background:**

Early in life, patients with cystic fibrosis (CF) are infected with microorganisms including bacteria and fungi, particularly *Pseudomonas aeruginosa* and *Aspergillus fumigatus*. Since recent research has identified the anti-inflammatory properties of statins (besides their lipid-lowering effects), we investigated the effect of fluvastatin on the production of the potent neutrophil chemoattractant chemokine, IL-8, in whole blood from CF patients, stimulated by *Pseudomonas aeruginosa* (LPS) and *Aspergillus fumigatus* (AFA) antigens.

**Results:**

Whole blood from adult patients with CF and from healthy volunteers was collected at the Rennes University Hospital (France). Blood was pretreated for 1 h with fluvastatin (0–300 µM) and incubated for 24 h with LPS (10 µg/mL) and/or AFA (diluted 1/200). IL-8 protein levels, quantified by ELISA, were increased in a concentration-dependent manner when cells were stimulated by LPS or AFA. Fluvastatin strongly decreased the levels of IL-8, in a concentration-dependent manner, in whole blood from CF patients. However, its inhibitory effect was decreased or absent in whole blood from healthy subjects. Furthermore, the inhibition induced by fluvastatin in CF whole blood was reversed in the presence of intermediates within the cholesterol biosynthesis pathway, mevalonate, farnesyl pyprophosphate or geranylgeranyl pyrophosphate that activate small GTPases by isoprenylation.

**Conclusions:**

For the first time, the inhibitory effects of fluvastatin on CF systemic inflammation may reveal the important therapeutic potential of statins in pathological conditions associated with the over-production of pro-inflammatory cytokines and chemokines as observed during the manifestation of CF. The anti-inflammatory effect could be related to the modulation of the prenylation of signalling proteins.

## Introduction

Cystic fibrosis (CF) is a multisystem disease during which systemic inflammation is more prolonged and pronounced than in many other chronic diseases. This inflammation results, in part, from an overproduction of pro-inflammatory cytokines from chronically infected lungs to the central circulation [1]. In particular, defects in cystic fibrosis transmembrane conductance regulator (CFTR) are associated with an increased production of interleukin (IL)-8, a potent neutrophil chemoattractant that stimulates a massive influx of neutrophils into the airways [2]. Interrupting the vicious cycle of infection and inflammation is effective in slowing the course of the disease and antibiotics have long been the staple of pulmonary therapy. Besides the anti-infective strategy, high dose ibuprofen, resulting in an anti-inflammatory effect, delayed the progression of lung disease but was responsible for adverse events that limited its therapeutic utility [3], [4]. A possible therapeutic intervention may be based on the neutralisation of cytokines and chemokines, such as tumor necrosis factor (TNF)-α, IL-1β and IL-8, with specific antibodies, receptor antagonists or inhibition of the intracellular signalling cascades that result in their production. However, alternative anti-inflammatory agents are necessary.

Statins are 3-hydroxy-3-methyl-glutaryl–coenzyme-A (HMGCoA) reductase inhibitors that have been used clinically as lipid lowering agents and have an established role in the treatment of atherosclerotic disease. However statins do more than just lower cholesterol [5]. Recent research has identified the anti-inflammatory properties of statins [6], [7]. These effects have been related to the prevention of the prenylation of signalling molecules with subsequent downregulation of pro-inflammatory gene expression [8]. Furthermore, it was shown that statins also reduce the stability of lipid raft formation with subsequent effects on immune activation and regulation [9]. Both effects resulted in reduced cytokines, chemokines and adhesion molecule expression [10]. The findings in a recent systemic review and meta-analysis show a possible association between statin treatment and reduced mortality rate after having been diagnosed with bacterial infections [11]. Recently a retrospective clinical analysis showed that simvastatin use was associated with a significant decrease in the rate of forced expiratory volume in one second (FEV_1_) and forced vital capacity (FVC) decline in smokers and non-smokers with airflow obstruction [12]. Indeed, recent studies have reported the therapeutic effect of statins in lung inflammatory diseases, such as in chronic obstructive pulmonary disease (COPD) [13], in asthma [14] and in acute lung injury [15], [16]. However, no study has evaluated the potential therapeutic interest of statins during CF.

Human immune cell lines only partially reflect the responses of primary cells. As blood of healthy donors and patients is easily accessible, numerous studies have been carried out employing isolated human peripheral blood cells. However, the highly sensitive leukocytes are easily affected and/or modulated by purification and isolation procedures. *Ex vivo* stimulation of whole blood appears to reflect closely what happens *in vivo*. Therefore the simple approach of employing human whole blood as an isolated organ is used to monitor immune function *ex vivo* [17], [18], [19].

The objective of this study was to investigate the anti-inflammatory effects of fluvastatin in reducing the circulating levels of IL-8 in an *ex vivo* model of human whole blood from patients with CF compared to healthy subjects. The fluvastatin inhibition was studied after stimulation with *Pseudomonas aeruginosa* and *Aspergillus fumigatus* antigens, alone or in combination. Finally, we examined whether fluvastatin inhibited whole blood IL-8 secretion through isoprenylation-dependent mechanisms. This work considers, for the first time, the important therapeutic potential of statins in pathological conditions associated with chronic inflammation such as those that occur in CF.

## Methods

### Ethics statement

This study was conducted according to the Good Clinical Practice guidelines and approved by the Ethical Committee of human subjects of the university hospital of Rennes, France (Ethics No. 07/32-649).

### Patients with CF

All patients included in this study gave their written informed consent. Twenty three stable adult patients with CF were recruited at the ‘*Centre de Ressources et de Compétences de la Mucoviscidose*’ (CRCM) of the Rennes University Hospital (France). Patients with CF considered for inclusion were Caucasian, 14 males and 9 females, aged between 18–44 years (mean age: 29±8). The CF diagnosis was based on typical clinical manifestations of the disease and confirmed by positive sweat tests and by CFTR gene mutation detection. The stable patients were defined by the absence of change in their symptoms in the 3 months prior to the study. All the patients with CF had medication at the time of blood collection, including azithromycin, aerosol of DNAse, inhaled corticosteroids and azole therapy. Patients who were chosen were not on oral corticosteroids therapy, as this may have impaired inflammatory stimulation at the time of blood collection, and this may have influenced results. The clinical features of these patients are described in Table 1.

**Table 1:** Demographic data expressed as numbers (number of patients in groups, genotype, medication, extrapulmonary diseases and infectious colonization, with percentage in brackets) or median (age, BMI, FEV_1_ and FCV, with range in brackets).

Parameters	Demographic data
Number of patients (males)	23 (14)
Median age in years	29 (18–44)
BMI (kg.m^−2^)	20 (15–24.5)
blood neutrophils count (×10^−9^/L)	5.5 (2.1–12.8)
blood monocytes count (×10^−9^/L)	0.64 (0.32–1.10)
CF genotype	
F508del/F508del	11 (48%)
F508del/other	8 (35%)
no F508del	4 (17%)
Pulmonary function tests	
FEV1, % predicted	68 (30–114)
FVC, % predicted	88 (46–125)
Medication	
Azithromycin	3 (14%)
DNAse aerosol	12 (55%)
Inhaled corticosteroids	15 (65%)
Azole therapy	9 (39%)
Extrapulmonary diseases	
Pancreatic insufficiency	17 (77%)
Liver disease	5 (23%)
Diabetes	6 (27%)
Infectious colonization	
*Pseudomonas aeruginosa*	16 (70%)
*Staphylococcus aureus*	19 (83%)
*Aspergillus fumigatus*	14 (61%)

All data were assessed at the time of sample collection. Blood neutrophils and monocytes count are not significantly different in CF patients group (one-way ANOVA, Kruskal-Wallis test).

**Abbreviations**: BMI, Body mass index; FEV1, Forced expiratory volume in one second; FVC, Forced vital capacity.

### Healthy volunteers

Healthy subjects included as controls were Caucasian, 4 males and 6 females, aged between 20–48 years (mean age: 31±11). They were healthy as determined by medical history, without personal history of CF or other significant illness, with a non-smoking status and were recruited from amongst the staff of respiratory department of the Rennes University Hospital (France).

### Experimental protocol

For each subject, 15 mL of whole blood was collected in heparin-tubes. Blood was aliquoted into 96-well microplates, under sterile conditions and incubated for 30 min at 37°C in an atmosphere of 5% CO_2_ and 95% air [19].

Pre-treatment with increasing concentrations of fluvastatin (0.01–300 µM) or vehicle were incubated for 1 h. At the end of this incubation period, *Pseudomonas aeruginosa* (LPS) and/or *Aspergillus fumigatus* (AFA) antigens were added into stimulated wells whereas unstimulated wells received a sterile saline vehicle only (NaCl 0.9%). To study the reversion of fluvastatin inhibition, mevalonate (416 µM) or the isoprenoids, farnesyl pyrophosphate (FPP, 10 µM) and geranylgeranyl pyrophosphate (GGPP, 10 µM) were added to fluvastatin during the 1 h pre-treatment. Reversion studies were performed with blood from five CF patients (2 with homozygous F508del mutation, 2 with heterozygous F508del mutation and 1 with other mutations) and five healthy volunteers from amongst the subjects recruited. After 24 h of stimulation the supernatants were gently transferred into polypropylene microtubes and stored at −80°C until the IL-8 assay was performed.

### Measurement of IL-8 release

IL-8 levels were quantified from diluted blood samples according to the manufacturer's instructions (PeproTech, TebuBio, Le Perray en Yvelines, France). A human IL-8 standard concentration-response curve was run on each microplate to allow determination of IL-8 levels in samples. IL-8 concentrations in each sample were determined using an automatic plate reader associated with Genesis software (LabSystems Spectrophotometer, Cambridge, UK) and data were expressed in ng/mL.

### Materials

Fluvastatin was acquired from Calbiochem (Merck Chemicals, Nottingham, UK), dissolved in sterile distilled water at a concentration of 5 mM and then diluted as required with sterile saline (NaCl 0.9%). LPS from *Pseudomonas aeruginosa*, mevalonate (mevalonic acid 5-phosphate trilithium salt hydrate), farnesyl pyrophosphate ammonium salt (FPP) and geranylgeranyl pyrophosphate ammonium salt (GGPP) were obtained from Sigma (St. Louis, MO, USA). FPP and GGPP in methanol∶ammonium solution (7∶3) were diluted in sterile saline. Soluble antigens from *Aspergillus fumigatus* (AFA) were provided from Bio-Rad Laboratories (Marnes-la-Coquette, France). They were dissolved in 1 mL sterile distilled water and then diluted using NaCl 0.9%.

### Statistical analysis

The number of subjects used in each group is stated in the respective figure legends. All graphs are expressed as mean ± SEM. IC_50_ values were calculated using regression analysis from dose-reponse curves (GraphPad Prism5 software, San Diego, USA). Statistical analyses were performed on the data and IC_50_ values using one-way ANOVA Kruskal-Wallis test or non-parametric Mann–Whitney test as appropriate (GraphPad Prism5 software). P<0.05 was considered to be statistically significant.

## Results

### Patients characteristics

Twenty three CF patients (14 male and 9 female subjects) were recruited for the study (Table 1). Lung function and microbiological testing were performed in all patients. According to the FEV_1_ values (% predicted), the majority of our patients (17/23) had mild to moderate lung disease (respectively FEV_1_ values≥55% of predicted values). Seventeen patients were affected by pancreatic insufficiency. Sixteen patients had microbiological evidence of *Pseudomonas aeruginosa* colonization and fourteen of *Aspergillus fumigatus*. The blood leukocytes count for CF patients was within normal range with a median number of 5.5×10^9^/L (range 2.1–12.8×10^9^/L) for neutrophils and of 0.64×10^9^/L (range 0.32–1.10×10^9^/L) for monocytes.

### Antigens from *Pseudomonas aeruginosa and Aspergillus fumigatus* increase IL-8 production

**Figure 1:**
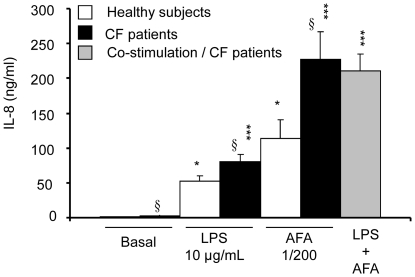
*Pseudomonas aeruginosa* and *Aspergillus fumigatus* antigens stimulation of IL-8 production. *Pseudomonas aeruginosa* (LPS, 10 µg/mL) and/or *Aspergillus fumigatus* (AFA, 1/200) antigens were added in the whole blood from 4 to 10 healthy subjects (open bars) and 23 CF patients (solid bars and grey bar for co-stimulation) for 24 h. After incubation, IL-8 release (ng/mL) was measured by ELISA. Data are shown as mean ± SEM. Mann and Whitney test: *P<0.05, *** P<0.0001 *vs* basal; § P<0.05 *vs* healthy patients.

Basal levels of IL-8 in whole blood from CF patients (2741±467 pg/mL, n = 23) were three times higher than those measured in blood from healthy volunteers (872±515 pg/mL, n = 10, p<0.05) (Fig. 1).

IL-8 levels were raised in a concentration-dependent manner when whole blood was stimulated with increasing concentrations of antigens from *Pseudomonas aeruginosa* (LPS) or from *Aspergillus fumigatus* (AFA) (not shown). The concentration of 10 µg/mL of LPS and the 1/200 dilution of AFA, which had induced a significant production of IL-8, were further used to assess the inhibitory effects of fluvastatin (Fig. 1). The LPS-induced level of IL-8 reached 51.8±8.2 ng/mL (n = 10) for healthy subjects and 80.2±9.9 ng/mL (n = 23) for CF patients. In whole blood from healthy subjects and CF patients, AFA induced the release of IL-8 to 113.6±27.2 ng/mL (n = 4) and 227.1±39.7 ng/mL (n = 23), respectively.

When both AFA and LPS were used in combination to stimulate whole blood, there were no additional increases of IL-8 production compared that of each antigen alone (Fig. 1).

### Fluvastatin inhibits IL-8 secretion in whole blood from CF patients

**Figure 2:**
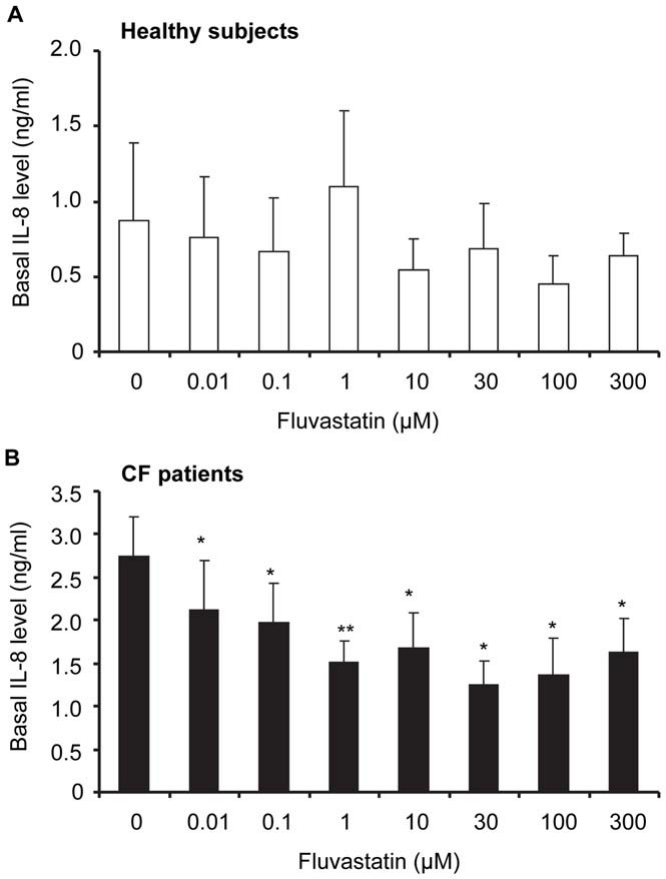
Fluvastatin inhibition of basal IL-8 production. Whole blood from healthy volunteers (**A**, n = 10) and from CF patients (**B**, n = 23) were treated with fluvastatin (0.01–300 µM, 1 h). After incubation, IL-8 release (ng/mL) was measured by ELISA. Data are shown as mean ± SEM. Mann and Whitney test: *P<0.05, ** P<0.001 *vs* without fluvastatin (0).

Without stimulation, fluvastatin from 0.01 µM significantly inhibited IL-8 basal levels in whole blood from CF patients but had no significant effect on whole blood from healthy subjects (Fig. 2). Maximal inhibition of IL-8 production by fluvastatin was observed at 30 µM with an efficacy value of 56.7±4.2% and an IC_50_ value of 13.1±5.3 µM (Table 2).

**Table 2:** IC_50_ values of fluvastatin (µM) calculated using regression analysis from dose-response curves (GraphPad Prism 5).

IL-8 release	healthy volunteers(n = 4–9)	CF patients(n = 17–22)
Basal	>300 µM	13.1±5.3 µM
LPS-induced	19.1±7.4 µM	4.6±1.0 µM**
AFA-induced	>300 µM	33.1±15.0 µM

Values are means ± SEM. Mann and Whitney test:

**P<0.001 vs healthy volunteers.

**Figure 3:**
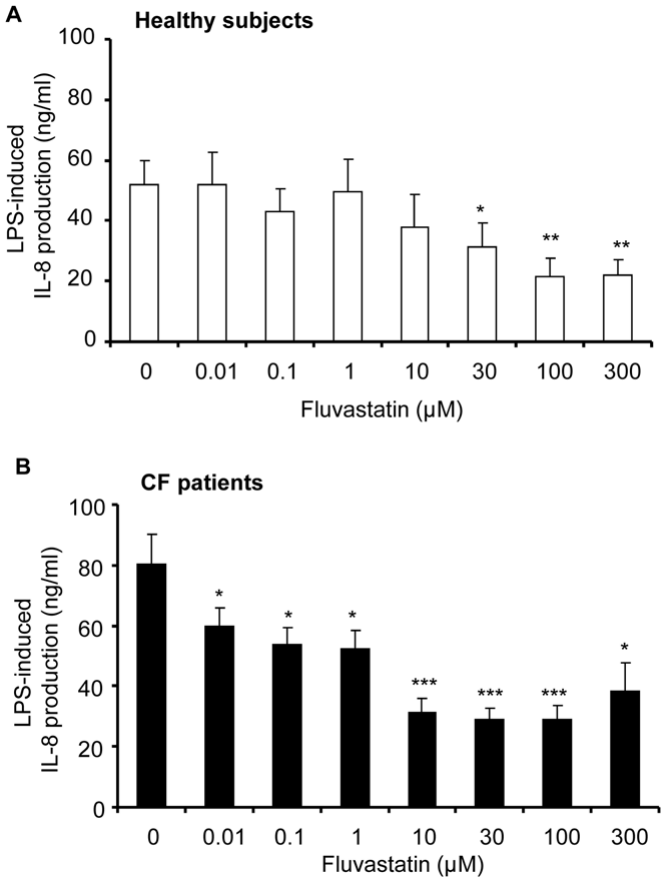
Fluvastatin inhibition of LPS-induced IL-8 production. Whole blood from healthy volunteers (**A**, n = 10) and from CF patients (**B**, n = 23) were pre-treated with fluvastatin (0.01–300 µM, 1 h) and incubated with *Pseudomonas aeruginosa* antigens (LPS, 10 µg/mL) for 24 h. After incubation, IL-8 release (ng/mL) was measured by ELISA. Data are shown as mean ± SEM. Mann and Whitney test: *P<0.05, ** P<0.001, *** P<0.0001 *vs* without fluvastatin (0).

From 0.01 µM, fluvastatin also decreased IL-8 production when whole blood from CF patients was stimulated with LPS (Fig. 3B) whereas a concentration of 30 µM was necessary to significantly inhibit IL-8 production in whole blood from healthy subjects (Fig. 3A). Fluvastatin was four times more potent in inhibiting LPS-induced IL-8 secretion in whole blood from CF patients than in healthy volunteers, with respective IC_50_ values being 4.6±1.0 µM and 19.1±7.4 µM (Table 2). However, the same maximal inhibition of LPS-induced IL-8 production was observed in CF patients and healthy volunteers (61.8±4.5% and 60.7±8.2% at 100 µM, respectively).

**Figure 4:**
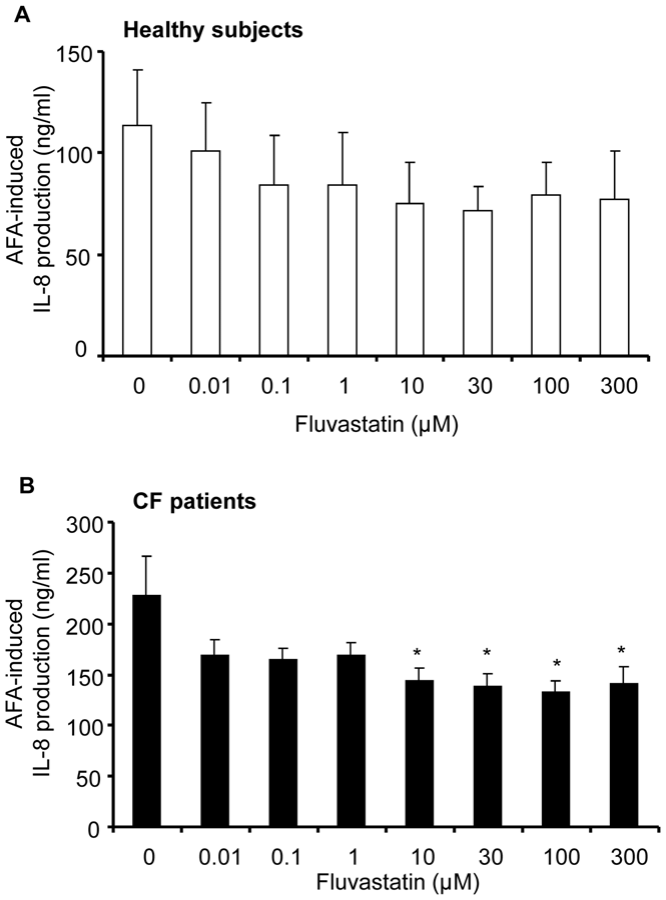
Fluvastatin inhibition of AFA-induced IL-8 production. Whole blood from healthy volunteers (**A**, n = 4) and from CF patients (**B**, n = 23) were treated with fluvastatin (0.01–300 µM, 1 h) and incubated with *Aspergillus fumigatus* antigens (AFA, 1/200) for 24 h. After incubation, IL-8 release (ng/mL) was measured by ELISA. Data are shown as mean ± SEM. Mann and Whitney test: *P<0.05 *vs* without fluvastatin (0).

When inflammatory stimulation was induced by AFA in whole blood from CF patients fluvastatin produce a significant inhibitory effect from 10 µM whereas no significant effect was observed in AFA-stimulated whole blood from healthy volunteers (Fig. 4). Fluvastatin was more potent in inhibiting IL-8 release in CF patients than in healthy subjects, with IC_50_ values being 33.1±15.0 µM and >300 µM respectively (Table 2). In this context, fluvastatin induced an unchanged maximal inhibition of IL-8 production in whole blood from CF patients and healthy subjects (27.1±9.1% and 27.5±4.4% at 100 µM respectively).

### Isoprenoids and mevalonate reverse fluvastatin inhibition

**Figure 5:**
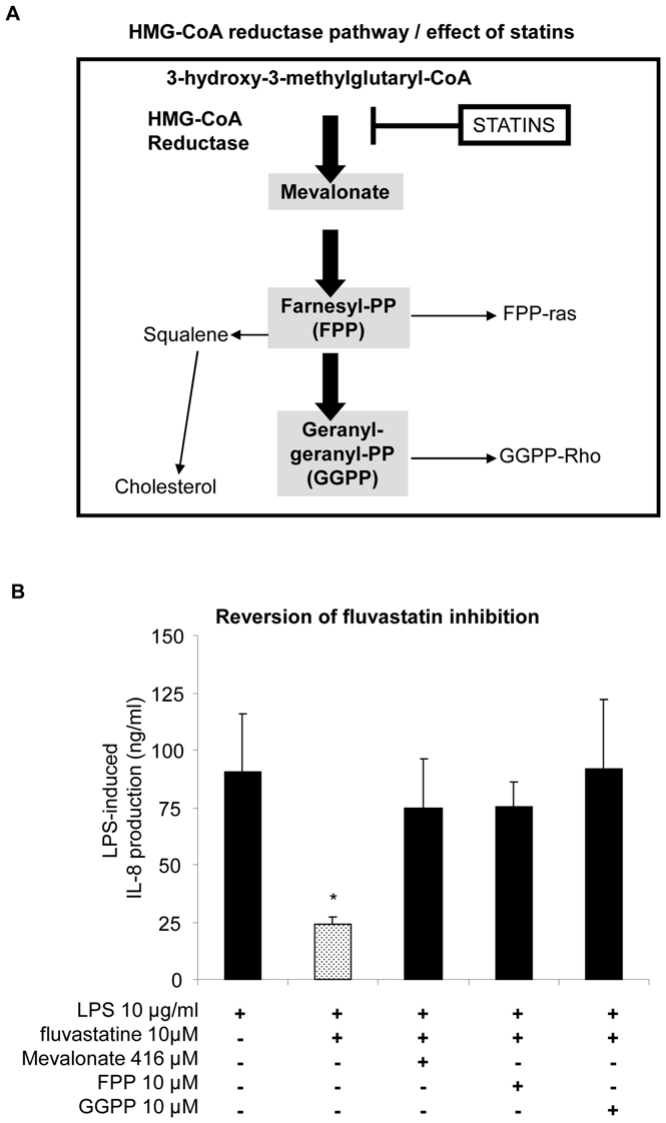
Reversion of fluvastatin inhibition by metabolite of 3-hydroxy-3-methylglutaryl-CoA (HMG-CoA). (**A**) Diagram summarizing HMG-CoA reductase pathway and the effect of statins. (**B**) Mevalonate (416 µM, 1 h) and isoprenoids, farnesyl pyrophosphate (FPP, 10 µM, 1 h) and geranylgeranyl pyrophosphate (GGPP, 10 µM, 1 h) reversed inhibitory effect of fluvastatin (10 µM, 1 h) on IL-8 production stimulated by LPS from *Pseudomonas aeruginosa* (LPS, 10 µg/ml, 24 h) in the whole blood from 5 CF patients. Data are shown as mean ± SEM. Mann and Whitney test: *P<0.05 *vs* LPS stimulation.

To characterize the statin target, individual components of the HMG-CoA reductase pathway, schematically shown in Fig. 5A, were combined with fluvastatin. The inhibitory effect of 10 µM fluvastatin on LPS-stimulated IL-8 release was reversed by mevalonate supplement (416 µM) or isoprenoids, FFP (10 µM) and GGPP (10 µM) supplements (Fig. 5B). By contrast, the inhibitory effect of fluvastatin was not reversed in the whole blood from healthy subjects (Fig. S1).

### CFTR mutation influences basal IL-8 release in whole blood from CF patients

**Figure 6:**
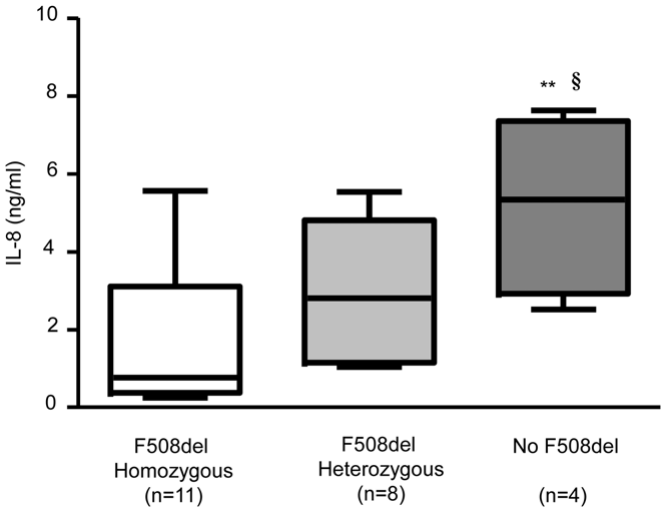
Basal IL-8 level and CF Mutations. CFTR mutations influence IL-8 basal secretion in whole blood from CF patients. Number of patients per group according to CFTR mutation is in brackets. Data are shown as mean ± SEM. Mann and Whitney test: ** P<0.001 *vs* F508del homozygous group; § P<0.05 *vs* F508del heterozygous group.

The concentration of IL-8 release in whole blood was dependent on CF mutations (Fig. 6). Significantly higher IL-8 levels were observed in the F508del mutation group (n = 4) compared to the groups with homozygous (n = 11) or heterozygous F508del mutation (n = 8). No significant difference between mutation groups was observed when whole blood was stimulated with LPS (83.11±11.9 ng/mL, 80.2±23.0 ng/mL and 71.9±18.7 ng/mL for homozygous, heterozygous F508del and no F508del mutation group, respectively). In contrast, regarding IL-8 secretion, the homozygous F508del group seemed to be more sensitive to AFA stimulation (274.9±78.1 ng/mL) than the heterozygous F508 group (178.08±36.1 ng/mL) or the F508del mutation group (193.67±17.9 ng/mL).

There was no observed difference on the inhibitory effect of fluvastatin between the various CFTR mutations groups. The only detectable difference between these 3 groups of patients, based on the CFTR mutation, was the frequency of the liver disease: 36% of the F508del homozygous group had liver disease *vs* 0% in the 2 other groups.

## Discussion

This study showed that *Pseudomonas aeruginosa* and *Aspergillus fumigatus* antigens induce IL-8 secretion in whole blood from CF patients and healthy volunteers. Basal and antigen-induced IL-8 releases were more inhibited by fluvastatin in CF patients than in healthy subjects and this effect was reversed in the presence of isoprenoids or mevalonate.

CF inflammation results from a complex combination of inflammatory processes due to the disease but also to recurrent infections. Bacteria and fungi are responsible for acute and chronic colonization and/or infection and thus represent important co-factors for local and systemic inflammation. *Aspergillus fumigatus*, the causative agent of allergic bronchopulmonary aspergillosis (ABPA), and *Pseudomonas aeruginosa*, particularly the mucoid strains, have been frequently isolated from the sputum of patients with CF [20]. When exposed to *Aspergillus fumigatus* or LPS, immune and pulmonary epithelial cells respond with increased synthesis and increased release of TNF-α, IL-1β, IL-8 and IL-6 [19], [21], [22]. These pro-inflammatory cytokines can further activate neutrophils and lymphocytes, initiating cellular injury and tissue damage [23]. Among them, IL-8 has an important role in neutrophil elastase-induced goblet cell metaplasia and the production of Muc5ac in airway epithelium [24]. In CF, elevated levels of IL-8 in sputum result in chronic infection, neutrophilic inflammation and progressive airway destruction. Furthermore whole blood IL-8 levels were related to the severity of CF [25]. As expected, when whole blood was stimulated with *Pseudomonas aeruginosa* or/and *Aspergillus fumigatus* antigens we observed a strong increase in IL-8 release that was not enhanced by co-stimulation. IL-8 levels were three times higher in CF patients than in healthy subjects confirming excessive systemic inflammation in CF. Enhanced expression of TLR4 in monocytes from children with clinically stable CF was also recently demonstrated. Toll-like receptors (TLRs), of which TLR2 and TLR4 on the cell surface of leukocytes are important in relation to host defense against pathogens trigger signalling pathways that activate NF-κB and transcription of pro-inflammatory cytokines and IL-8 [25]. A significant increase in IL-8 in the whole blood assay is an interesting finding, with regard to previous research. The few studies performed were not able to provide evidence of a significant correlation between serum IL-8 and clinical status of the patient [26], [27], and Kronborg *et al.* (1993) [28] found only low or non-detectable levels of circulating cytokines in plasma. The differences between those results and our findings can be explained by the fact that cytokines are active compounds in plasma, rapidly bound to their soluble receptor and then neutralized [29], [30]. Taking this into account, plasma is not a good source for measuring the host's propensity to secrete cytokines. This problem can be overcome by using the whole blood assay where inhibitory factors are reduced by dilution with saline and cultured mononuclear cells constantly secrete cytokines in the supernatant. The immune cells are present in natural ratios and can interact with each other allowing considering whole blood as an isolated organ. As no isolation procedure beyond the drawing of blood is required, the assay is characterized by few preparation artifacts and standardized performance. Volunteers are not exposed directly to potentially hazardous agents such as lipopolysaccharide (LPS) as in the case of human experimental endotoxemia, yet information about immune modulation under conditions of leukocyte activation or overactivation can be obtained [31]. The advantage of using whole blood is the exclusion of secondary changes of the cells in the intensely inflamed environment of the CF airway that might have an influence on final results. Apparently, the human organism has to control the responsiveness of its leukocytes very carefully: any hyporesponsiveness will result in infectious complications and any hyperresponsiveness in inflammatory disorders. A major concern against whole blood models is the interindividual variation in leukocyte numbers. In fact, in healthy donors the normal range of leukocyte numbers is within a fairly tight window, between 4.5 and 11×10^9^/L. As previously observed [32], the blood leukocytes count for stable CF patients was within normal range.

Statins are a class of cholesterol-lowering drugs that decrease the mortality of cardiovascular diseases [33]. However their therapeutic potential also relies on anti-inflammatory effects. Several chronic lung inflammatory diseases seem to be improved by statins, as reported in asthma [14], COPD [13], acute lung injury, sepsis and infection [16], [34], [35]. There are also reports that the statins can inhibit T and B cell activation, prevent inflammatory responses and block activation of leukocyte subsets [10], [36], [37], [38], [39]. Furthermore, protection by simvastatin includes the inhibition of host cell invasion by *Staphylococcus aureus* due in part to depletion of isoprenoid intermediates within the cholesterol biosynthesis pathway [40]. In addition, the modulation of neutrophilic apoptosis, as observed under statin treatment [41], may prove beneficial in chronic inflammatory lung diseases such as CF, during which neutrophil numbers are significantly increased in airways [2]. Neutrophilia associated with acute lung injury in a mouse model was markedly reduced after treatment with lovastatin [42]. Mucus hypersecretion in the respiratory tract also contributes to airway inflammation, particularly during CF. Ou *et al.* (2008) [43] have demonstrated that simvastatin attenuates airway mucus hypersecretion and pulmonary inflammatory damage induced by LPS, in rats. Also, lovastatin has recently been shown to inhibit human alveolar epithelial production of IL-8 [44]. Furthermore, the outcomes in lung transplantation were compared between patients treated with statins for hyperlipidaemia and untreated controls. Acute rejection was less frequent; bronchoalveolar lavage showed lower total cellularity as well as lower numbers of neutrophils and lymphocytes, and survival was 91% compared with 54% in controls [45]. This raises the intriguing possibilities of the therapeutic interest of statin in lung transplanted CF patients. However at present, no study has investigated this potential therapeutic interest of statins in CF.

It has recently emerged that different families of statins may have various anti-inflammatory properties [34]. Kiener *et al.* (2001) [46] showed that lipophilic statins such as atorvastatin, simvastatin and fluvastatin have a much greater effect on the inflammatory response in human and mouse models than the hydrophilic, pravastatin. Furthermore fluvastatin has a very potent hypocholesterolemic effect with fewer adverse effects than other HMG-CoA reductase inhibitors [47]. In addition to its lipophilic and anti-inflammatory properties, fluvastatin also has the advantage of not requiring activation prior to its use for *in vitro* studies and it does not present toxicity in leukocytes (Fig. S2) [47]. In our study, fluvastatin (from 0.01 µM) inhibits IL-8 release from whole blood cells of CF patients in different conditions including in unstimulated cells, those stimulated by antigens from *Pseudomonas aeruginosa* or, with lesser effect, by soluble antigens from *Aspergillus fumigatus*. The efficacy of fluvastatin was unchanged in whole blood from all subjects whereas its IC_50_ was four times lesser in CF patients than in healthy volunteers. This suggests an improved pharmacological profile in terms of *in vitro* efficacy and potency, and thus a potential increased therapeutic index. This attenuation of LPS-induced inflammation was previously observed with simvastatin in a randomized double-blind placebo-controlled clinical study involving healthy volunteers challenged with LPS aerosols [48]. However, we have observed a better effect of fluvastatin in whole blood from CF patients compared with healthy volunteers. Indeed, while TLRs expression is enhanced in CF monocytes [25], fluvastatin dose-dependently inhibits monocyte TLR4 and TLR2 expressions in whole blood from patients with chronic heart failure [49].

Besides the reduction of cholesterol biosynthesis, through competitive inhibition of the enzyme HMG-CoA reductase, the current hypothesis for the pleiotropic effect favours the inhibition of isoprenoid biosynthesis as the likely mechanism. The mevalonate synthetic pathway mediated by HMG-CoA reductase is crucial for the biosynthesis of isoprenoids, which are essential for normal cellular inflammatory signalling. Farnesylpyrophosphate is a later intermediate on this pathway and serves as a precursor for the synthesis of various isoprenoids - for example, geranylgeranyl or farnesyl groups - which prenylate proteins through covalent links [8]. These can anchor prenylated proteins to lipid rafts and many of them play important roles in the regulation of cell growth, cell secretion, and signal transduction. Thus, by inhibiting prenylation, statins should affect many cell processes involved in inflammation. In our model of whole blood from CF patients, isoprenoids reversed the inhibition of LPS-stimulated IL-8 production suggesting that fluvastatin acts by preventing the prenylation of signalling molecules, such as rho-A, ras or rac, implicated in IL-8 signalling [50]. Increased rhoA expression and activation was demonstrated in CF cells [51] and may explain that reversion is only observed in whole blood from CF patients and not in healthy subjects.

Finally, we have observed that patients with the non-F508del mutation show higher basal levels of IL-8 than homo- or heterozygous F508del mutation. Little research has studied the phenotypic consequences of CFTR mutations [52], [53]. Our results, which should be confirmed on a larger patient cohort, suggest that the CFTR genotype may be involved in whole blood IL-8 release related to systemic inflammation in CF patients. However, we could not find a significant difference in IL-8 production after stimulation with LPS or AFA in any of the CF mutation groups. Therefore enhanced IL-8 secretion after induced acute inflammation in whole blood from CF patients may not related to CFTR genotype.

In conclusion, fluvastatin inhibits basal *Pseudomonas aeruginosa* and *Aspergillus fumigatu*s antigen-induced IL-8 secretion in whole blood from CF patients through an isoprenylation-dependent mechanism. The inhibitory effects of fluvastatin on systemic inflammation may reveal the important therapeutic potential of statins in various pathological conditions associated with the over-production of pro-inflammatory cytokines and chemokines as observed in CF.

## Supporting Information

Figure S1 **No reversion of fluvastatin inhibition by metabolite of HMG-CoA in whole blood from healthy volunteers.** Mevalonate (416 µM, 1 h) and isoprenoids, farnesyl pyrophosphate (FPP, 10 µM, 1 h) and geranylgeranyl pyrophosphate (GGPP, 10 µM, 1 h) have not reversed inhibitory effect of fluvastatin (10 µM, 1 h) on IL-8 production stimulated by LPS from *Pseudomonas aeruginosa* (LPS, 10 µg/ml, 24 h) in the whole blood from 5 healthy subjects. Data are shown as mean ± SEM.
(TIF)

Figure S2 **MTT assay was used to determine viability of cells treated with fluvastatine.** Human leukocytes from healthy subjects obtained fom Ficoll gradient were seeded into 24-well plates at a density of 4×10^6^ per well and treated with fluvastatin at 0.01 to 100 µM (1 h), alone or in the presence of *Pseudomonas aeruginosa* antigens (LPS, 10 µg/ml, 24 h). Wells with vehicle (NaCl 0.9%) were used as negative control. At 1 h before each of the desired time points, 100 µl of MTT solution (5 mg/ml in PBS, 3-(4,5-dimethylthiazol-2-yl)-2,5-diphenyltetrazolium bromide, Sigma, La Verpillière, France) was added into each well and cells were incubated at 37°C and 5% CO_2_ for another 2 h. The medium was removed and 100 µl of DMSO was added into each well. The plate was gently rotated on an orbital shaker for 10 min to completely dissolve the precipitation. The absorbance was detected at 570 nm with a microplate reader associated with Genesis software (LabSystems Spectrophotometer, Cambridge, UK).
(TIF)
